# Psoriasis in Obese Patients: Pathophysiological Interactions, Clinical Consequences, and Therapeutic Implications

**DOI:** 10.3390/jcm15114302

**Published:** 2026-06-02

**Authors:** Gustavo Almeida-Silva, Joana Antunes, João Ferreira, Paulo Filipe

**Affiliations:** 1 Department of Dermatology, Hospital de Santa Maria, Unidade Local de Saúde Santa Maria, 1649-035 Lisbon, Portugal; gustavofasilva@gmail.com (G.A.-S.);; 2 Dermatology University Clinic, Faculty of Medicine, University of Lisbon, 1649-028 Lisbon, Portugal

**Keywords:** psoriasis, obesity, IL-23, biologics, adipokines, systemic inflammation, biologic therapy, metabolic syndrome

## Abstract

**Background/Objectives**: Psoriasis is a chronic immune-mediated inflammatory disease increasingly recognized as a systemic disorder associated with significant metabolic and cardiovascular comorbidities. Among these, obesity (defined as BMI > 30 kg/m^2^) plays a pivotal role, acting both as a risk factor for psoriasis development and as a modifier of disease severity, clinical phenotype, and therapeutic response. The relationship between psoriasis and obesity is bidirectional and sustained by shared inflammatory and metabolic pathways. This review aims to provide a comprehensive and updated synthesis of the epidemiological association between psoriasis and obesity, to elucidate the underlying pathophysiological mechanisms, and to discuss the clinical and therapeutic implications of excess body weight in psoriasis management. **Methods**: A narrative review of the literature was conducted, including epidemiological studies, mechanistic research, clinical trials, and real-world evidence addressing the interplay between psoriasis and obesity. Relevant data were identified from peer-reviewed publications focusing on inflammatory pathways, metabolic dysfunction, cardiovascular risk, and treatment outcomes in obese patients with psoriasis. The graphical figures included in this manuscript were created with the assistance of a large language model–based image-generation tool, ChatGPT-5 by OpenAI, using author-defined prompts. The prompts requested schematic medical illustrations summarizing the pathophysiological links between obesity and psoriasis, including adipose tissue dysfunction, adipokine imbalance, systemic inflammation, and activation of the IL-23/Th17 axis. For the therapeutic algorithm, the prompt requested a stepwise clinical flowchart for obese patients with psoriasis, including BMI assessment, comorbidity screening, universal weight-management measures, psoriasis severity stratification, obesity-adapted biologic selection, and management of suboptimal response. The generated images were subsequently reviewed, edited, and approved by the authors to ensure scientific accuracy, clarity, and consistency with the manuscript content. **Results**: Epidemiological evidence consistently demonstrates a higher prevalence of obesity among patients with psoriasis, with obesity independently associated with increased disease severity. Shared mechanisms include adipose tissue–driven cytokine production, dysregulated adipokine secretion, insulin resistance, endothelial dysfunction, and activation of the IL-23/Th17 axis, collectively contributing to systemic inflammation and accelerated atherogenesis. Obesity negatively impacts the efficacy, pharmacokinetics, and long-term drug survival of conventional systemic agents and biologic therapies, leading to suboptimal clinical outcomes. **Conclusions**: Obesity is a key determinant of psoriasis burden, influencing disease expression, comorbidities, and therapeutic response. Integrating weight reduction strategies into personalized psoriasis management may improve both dermatological outcomes and overall cardiometabolic health, supporting a holistic approach to patient care.

## 1. Introduction

**Figure 1:**
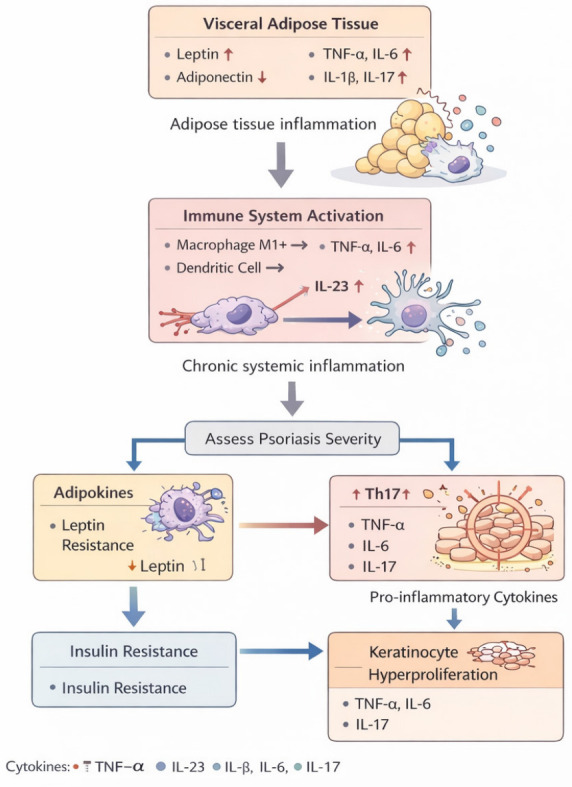
Pathophysiology of inflammation in obesity and psoriasis: visceral adipose tissue dysfunction leads to increased secretion of pro-inflammatory adipokines (e.g., leptin) and inflammatory cytokines (TNF-alpha; IL-6, IL-23, IL-1β, IL-17), promoting chronic systemic inflammation; this state drives insulin resistance and enhances IL-23-mediated Th17 differentiation.

Psoriasis is a chronic, immune-mediated inflammatory skin disease affecting approximately 2–3% of the global population worldwide [1]. While historically regarded as a disease limited to the skin and joints, psoriasis is now recognized as a systemic inflammatory disorder associated with multiple extracutaneous comorbidities, particularly cardiometabolic diseases [2,3]. Large epidemiological studies have consistently demonstrated that patients with psoriasis have an increased prevalence of obesity, type 2 diabetes mellitus, dyslipidemia, hypertension, non-alcoholic fatty liver disease (NAFLD), and major adverse cardiovascular events [2,3]. Among these comorbidities, obesity occupies a pivotal position, acting as both a risk factor for psoriasis development and a determinant of disease severity and therapeutic response [4,5]. The association between psoriasis and obesity is complex and bidirectional: obesity predisposes individuals to psoriasis through chronic low-grade inflammation, while psoriasis may promote weight gain due to physical inactivity, psychosocial burden, and systemic inflammatory effects [2,6]—see Figure 1. Understanding this interplay is essential for optimizing long-term outcomes in patients with psoriasis.

Body mass index (BMI) is calculated as weight in kilograms divided by the square of height in meters (kg/m^2^) and remains the most widely used metric for categorizing body weight in clinical and epidemiological studies. According to the World Health Organization (WHO) classification, overweight is defined as a BMI between 25.0 and 29.9 kg/m^2^, while obesity is defined as a BMI of ≥30.0 kg/m^2^. Obesity is a serious chronic disease characterized by excessive accumulation of body fat to the extent that it may have a negative effect on health. Obesity is further subclassified into class I (30.0–34.9 kg/m^2^), class II (35.0–39.9 kg/m^2^), and class III or severe obesity (≥40.0 kg/m^2^). Increasing BMI categories are associated with progressively higher systemic inflammatory burden, greater psoriasis severity, reduced therapeutic response, and increased cardiometabolic risk.

Multiple population-based cohort and case–control studies have identified obesity as an independent risk factor for the development of psoriasis [4,5,6]. Prospective data demonstrate a dose-dependent relationship between body mass index (BMI) and psoriasis incidence, with progressively higher risk observed at increasing BMI categories [5]. Measures of central adiposity, including waist circumference and waist-to-hip ratio, appear to be even stronger predictors of psoriasis risk than BMI alone, highlighting the pathogenic relevance of visceral fat accumulation [5].

Meta-analyses indicate that obese individuals have approximately a 1.5–2-fold increased risk of developing psoriasis compared with normal-weight individuals, even after adjustment for smoking, alcohol consumption, and physical activity [4]. These findings support a causal role for obesity in psoriasis pathogenesis rather than a mere association.

The relationship between psoriasis and obesity is bidirectional [2,6]. Patients with psoriasis may be predisposed to weight gain due to reduced physical activity related to joint involvement, pain, or social stigma associated with visible skin lesions [6]. In addition, depression and anxiety—frequent comorbidities in psoriasis—may promote unhealthy dietary behaviors and sedentary lifestyles [2]. Chronic systemic inflammation itself may further alter energy metabolism and adipose tissue function, thereby reinforcing weight gain [2].

Obesity has been consistently associated with increased psoriasis severity [4,7]. Obese patients are more likely to present with moderate-to-severe disease, higher Psoriasis Area and Severity Index (PASI) scores, greater body surface area involvement, and increased need for systemic therapy [4,7]. Obesity has also been linked to earlier disease onset and a higher prevalence of psoriatic arthritis [3].

A randomized clinical study by Ali Ismail et al. demonstrated that a 12-week structured low-calorie diet combined with increased physical activity significantly improved psoriasis severity (assessed by PASI), metabolic parameters (BMI, triglycerides, liver enzymes), and quality of life in class I obese men with chronic plaque psoriasis and non-alcoholic fatty liver disease, compared with pharmacological therapy alone. In a complementary evidence synthesis, Morrow et al. conducted a systematic review and meta-analysis of weight-loss interventions in adults with psoriasis, finding that weight reduction through diet, exercise, or pharmacotherapy produced greater reductions in PASI scores and improved Dermatology Life Quality Index outcomes, supporting the incorporation of weight-loss strategies into routine management of psoriasis [8,9].

An Italian multicenter study of 114 overweight/obese patients with moderate-to-severe plaque psoriasis (mean BMI 32.1 kg/m^2^) found that although almost all patients (98.2%) were aware of the negative impact of obesity on general health, awareness of its specific impact on psoriasis was substantially lower: 42.7% were unaware of its effect on disease severity and 60% were unaware of its impact on treatment response. The study highlights a critical educational gap regarding obesity’s role in psoriasis outcomes, while also demonstrating strong patient readiness to engage in weight-loss interventions, supporting routine integration of nutritional counselling into psoriasis management [10].

Obesity should be regarded as a complex, multicomponent condition in which metabolic, nutritional, inflammatory, behavioural and psychosocial factors that may converge to aggravate psoriasis. In this context, vitamin D deficiency may represent an additional modifiable factor. Vitamin D exerts immunomodulatory and anti-inflammatory effects, contributes to epidermal barrier integrity and keratinocyte homeostasis, and has been linked to psoriasis severity; lower 25-hydroxyvitamin D levels may therefore increase susceptibility to disease exacerbation when additional triggers are present. In a retrospective case–control study of psoriasis patients following COVID-19 vaccination, those who developed flares had significantly lower vitamin D levels than those without exacerbation, and flare rates were higher among patients with vitamin D insufficiency or deficiency [11]. Moreover, obesity in psoriasis is closely intertwined with psychosocial burden. A systematic review found that higher BMI in adults with psoriasis was positively associated with depression, anxiety, impaired quality of life, poorer sleep, sexual dysfunction and difficulties in daily functioning, although most available data were cross-sectional. These findings support a broader biopsychosocial interpretation of obesity in psoriasis, in which nutritional deficits, psychological stress, depression and behavioural barriers may amplify systemic inflammation, reduce adherence to lifestyle interventions and contribute to recurrent disease flares [12].

## 2. Pathophysiological Links Between Psoriasis and Obesity

Adipose tissue is now recognized as an active immuno-endocrine organ rather than a passive energy reservoir [2]. In obesity, adipocyte hypertrophy leads to hypoxia, oxidative stress, and adipocyte apoptosis, which in turn promote infiltration of pro-inflammatory immune cells, particularly macrophages [2]. This process results in chronic low-grade systemic inflammation characterized by increased production of cytokines such as tumor necrosis factor-α (TNF-α), interleukin (IL)-6, IL-1β, and IL-17 [2]. These mediators are central to psoriasis pathogenesis and contribute directly to keratinocyte hyperproliferation, angiogenesis, and sustained immune activation within psoriatic plaques [1].

Adipokines are bioactive molecules secreted by adipose tissue that modulate metabolic and immune pathways [7]. Obesity is associated with a profound imbalance in adipokine production, which directly influences psoriatic inflammation [7]. Leptin levels are elevated in obese individuals and correlate positively with psoriasis severity [7]. Leptin promotes Th1 and Th17 differentiation, enhances pro-inflammatory cytokine production, stimulates keratinocyte proliferation, and suppresses regulatory T-cell activity [7]. In contrast, adiponectin—an adipokine with anti-inflammatory and insulin-sensitizing properties—is reduced in obesity and inversely correlated with PASI scores [7]. Reduced adiponectin levels may therefore contribute to both cutaneous inflammation and metabolic dysfunction. Other adipokines, including resistin and visfatin, have also been implicated in psoriasis pathophysiology, further supporting the role of adipose tissue–driven immune dysregulation [7].

The IL-23/Th17 axis is a central pathogenic pathway in psoriasis and is also activated in obesity [2,3]. Adipose tissue–derived cytokines promote Th17 polarization and IL-17 production, which drives keratinocyte activation, neutrophil recruitment, and epidermal hyperplasia [1]. IL-17 and IL-23 further contribute to insulin resistance, endothelial dysfunction, and atherogenesis, providing a mechanistic link between psoriasis, obesity, and cardiovascular disease [2,3].

A critical distinction must be made between metabolic disease–associated obesity and genetically defined obesity, as these entities differ substantially in their immunometabolic consequences. Metabolic obesity, typically driven by environmental factors such as caloric excess, sedentary lifestyle, and high-fat diet (HFD), is characterized by chronic low-grade inflammation, adipokine dysregulation, insulin resistance, and activation of pro-inflammatory pathways.

This inflammatory phenotype is highly relevant to psoriasis pathogenesis, as it promotes activation of the IL-23/Th17 axis, enhances keratinocyte proliferation, and sustains systemic immune activation. In contrast, genetically defined obesity does not necessarily recapitulate this pro-inflammatory state. Experimental evidence, including murine models, suggests that obesity in the absence of metabolic inflammation does not exacerbate psoriasiform dermatitis [13].

A 2019 Cochrane review found that dietary intervention with strict caloric restriction may lead to 75% or greater improvement in PASI scores (RR 1.66, 95% CI 1.07–2.58) and probably improves quality of life and reduces BMI in obese patients [14].

These benefits are maintained for up to one year and work synergistically with biologics, methotrexate, cyclosporine, and phototherapy [15].

These findings indicate that metabolic dysfunction rather than adiposity per se is the key pathogenic driver linking obesity to psoriasis.

Collectively, these data reinforce the concept that diet-induced metabolic inflammation is a major determinant of psoriasis severity, highlighting the importance of nutritional interventions in disease management.

## 3. Clinical Manifestations of Psoriasis and Comorbidities in Obese Patients

Obese patients with psoriasis frequently exhibit more extensive and severe disease [4,7]. Certain phenotypes appear to be overrepresented, including inverse psoriasis affecting intertriginous areas, where friction, maceration, and local inflammation exacerbate lesions [3]. Mechanical stress and Koebnerization may further influence lesion distribution in obese individuals. Obesity is a significant risk factor for the development and progression of psoriatic arthritis [3]. Obese patients are more likely to develop joint involvement and tend to present with higher disease activity scores, increased functional impairment, and poorer therapeutic responses [3]. Excess mechanical load, systemic inflammation, and adipokine-mediated immune activation are all implicated in joint pathology.

The coexistence of psoriasis and obesity markedly amplifies cardiometabolic risk [2,3]. Obese patients with psoriasis have higher prevalence of hypertension, dyslipidemia, insulin resistance, NAFLD, and major adverse cardiovascular events compared with non-obese psoriatic patients [2,3]. Chronic systemic inflammation acts as a shared pathogenic denominator, accelerating endothelial dysfunction and atherogenesis [2].

## 4. Impact of Obesity on Psoriasis Treatment

Obesity adversely affects the efficacy and safety of conventional systemic therapies [16,17]. Methotrexate is associated with increased risk of hepatotoxicity in obese patients, particularly in the presence of NAFLD [3,16]. Cyclosporine dosing is challenging due to altered pharmacokinetics and increased cardiovascular risk, while acitretin may exacerbate dyslipidemia [3,16,17].

Methotrexate shows reduced efficacy in obese patients with psoriasis. A retrospective cohort study of Asian patients found that BMI ≥ 25 kg/m^2^, female sex, and abdominal obesity were associated with decreased response to methotrexate in univariate analysis, with male sex being the only significant protective factor in multivariate analysis [18].

The AAD-NPF guidelines emphasize that patients with obesity, diabetes, and hyperlipidemia are at increased risk for methotrexate-induced hepatotoxicity due to higher prevalence of nonalcoholic fatty liver disease, which is also a common psoriasis comorbidity that can be aggravated by methotrexate. A 2025 expert consensus panel confirmed that obesity may decrease efficacy and potentiate side effects of conventional therapies including methotrexate [16,17].

Cyclosporine demonstrates a unique pharmacokinetic benefit when dosed by actual body weight in obese patients. The AAD-NPF guidelines specifically state that obese patients are more effectively treated when dosed according to their actual body weight [16].

A landmark randomized controlled trial by Gisondi et al. (2008) [19] demonstrated that weight loss significantly enhances cyclosporine efficacy in obese patients: 61 obese patients (BMI >30) with moderate-to-severe psoriasis were randomized to receive cyclosporine 2.5 mg/kg/day with or without a low-calorie diet. At 24 weeks, the intervention group lost 7.0% ± 3.5% of body weight and 66.7% achieved PASI 75, compared to only 29.0% in the control group (*p* < 0.001). The combination of cyclosporine with dietary weight reduction appears particularly effective for obese patients.

Acitretin presents significant safety concerns in obese patients due to metabolic complications. The AAD-NPF guidelines note that the risk of acitretin-induced hypertriglyceridemia is higher in patients with obesity, diabetes mellitus, or excessive alcohol intake—all conditions commonly seen in the psoriatic population. Hyperlipidemia occurs in 25–50% of patients on acitretin and is the most common laboratory abnormality, with rare cases of severe hypertriglyceridemia leading to fatal pancreatitis. Acitretin efficacy is generally lower than other systemic therapies regardless of weight status, with only 23% achieving PASI 75 at 8 weeks with 50 mg/day dosing. No specific studies were identified examining acitretin efficacy stratified by obesity status, though the 2025 expert consensus confirmed obesity may potentiate side effects of conventional therapies [16,17].

A 2023 BADBIR cohort study of 5430 patients found that acitretin had the lowest effectiveness (21% achieving aPASI ≤2) compared to cyclosporine (34%), FAEs (29%), and methotrexate (32%), with comorbidities being a risk factor for treatment ineffectiveness [20].

Biologic Therapies: Impact of Obesity on Clinical Response and Drug Survival

Multiple studies have demonstrated reduced clinical response to biologic therapies in obese patients, particularly with fixed-dose agents [21]. Higher body weight is associated with lower serum drug concentrations, reduced rates of PASI90 and PASI100 responses, and increased likelihood of treatment discontinuation [21]. Although weight-based dosing regimens may partially mitigate this effect, obesity remains an independent predictor of reduced drug survival [21].

Most psoriasis biologics are administered using fixed dosing, and higher body weight is associated with larger volume of distribution and faster clearance, which can lower drug exposure and contribute to reduced effectiveness in obese patients [22,23]. Evidence across registries and real-world cohorts consistently identifies obesity (or higher BMI/weight) as a clinically relevant predictor of lower PASI responses, higher discontinuation due to ineffectiveness, and reduced drug survival, although the magnitude of this effect varies by biologic class and molecule [21,22,24,25].

### 4.1. Anti-TNF Agents (Etanercept, Adalimumab, Infliximab, Certolizumab Pegol)

Across multiple observational studies, elevated BMI has been associated with attenuated response to anti-TNF therapy in psoriasis, likely reflecting exposure–response effects and obesity-driven systemic inflammation [22,24]. However, results are heterogeneous, and some datasets report minimal or no impact of BMI on specific anti-TNF agents, especially in long-term real-world settings [25].

Adalimumab: Registry analyses show that discontinuation due to ineffectiveness is influenced by multiple patient factors, and obesity has been repeatedly evaluated as a contributor to reduced persistence and effectiveness in practice [25]. In some real-world cohorts, BMI did not independently impair long-term adalimumab effectiveness, highlighting between-study variability and the confounding role of disease duration, prior biologic exposure, and comorbidity burden.

Etanercept: Similar to adalimumab, evidence suggests that higher BMI can reduce response probabilities in some cohorts, but findings are inconsistent across studies and endpoints [22,24].

Infliximab (weight-based dosing): Infliximab is administered per kg body weight, and subgroup analyses of clinical trial data suggest comparable short-term efficacy across BMI categories, supporting the concept that weight-adjusted dosing may mitigate exposure-related loss of response observed with fixed-dose agents [22,24]. Nevertheless, real-world dosing may drift below target mg/kg in severe obesity, potentially compromising exposure if dose intensity is not maintained [22,24].

Certolizumab pegol: Data specifically addressing BMI and certolizumab outcomes in cutaneous psoriasis are comparatively limited relative to other anti-TNF agents, and most “obesity–response” conclusions are extrapolated from class effects and broader real-world analyses [22,24].

Clinical implication: In patients with severe obesity and suboptimal response to fixed-dose biologics, a weight-based option (infliximab) or switching to a highly potent class with robust efficacy across subgroups (e.g., IL-17/IL-23 blockade) is commonly considered, alongside structured weight management [19,21,22,24,26].

### 4.2. Anti-IL-12/23 (Ustekinumab)

Ustekinumab is the only widely used psoriasis biologic with label-embedded weight stratification, with higher dosing recommended above a weight threshold, reflecting known exposure–response relationships [22,24,25]. Real-world datasets and drug-survival studies identify BMI/weight as a clinically relevant covariate, with several analyses reporting reduced persistence or response at higher BMI despite dose adjustments [25]. In comparative registry work, effectiveness-related discontinuation varies across biologics, and patient-level predictors (including weight/BMI) contribute to these differences [25].

Clinical implication: In obese patients receiving ustekinumab, ensure appropriate weight-aligned dosing, reassess early for suboptimal response, and consider class switching if PASI targets are not achieved [22,25].

### 4.3. Anti-IL-17

Several cohorts suggest that obesity can be associated with lower PASI responses to IL-17 pathway inhibitors, particularly for stringent endpoints (PASI90/100), although high efficacy is still achievable in many obese patients [21,25,27].

Secukinumab and ixekizumab: Real-world analyses have reported that BMI ≥ 30 kg/m^2^ may be associated with lower likelihood of optimal response in some populations, while other datasets show less pronounced effects, underscoring the importance of context (baseline severity, prior biologic exposure, adherence, and comorbidity) [21,25].

Brodalumab: Data specifically examining obesity suggest that brodalumab can maintain clinically meaningful effectiveness across BMI strata, with some reports showing broadly comparable long-term outcomes between obese and non-obese groups, although short-term differences may still be observed depending on the endpoint and cohort characteristics [21,27].

Bimekizumab: Post-hoc subgroup analyses of phase 3/3b programs include BMI strata up to severe obesity and provide structured evidence that high levels of skin clearance can be sustained across weight/BMI subgroups [28]. Real-world studies focused on obese cohorts further support effectiveness in difficult-to-treat disease, albeit with expected limitations of observational designs [21,28].

For obese patients needing rapid and deep clearance, IL-17 blockade remains a high-potency option; however, clinicians should monitor early response and consider switching within or across classes if PASI90/100 targets are not met [21,28].

In clinical practice, insufficient response to anti-IL17 drugs in obese patients is often tackled with dose escalation beyond standard labeled dosing schedules. There are no randomized clinical trials to substantiate these dose adjustements. Current evidence suggests that while escalation of secukinumab dosing occasionally improves outcomes in select patients (especially those with higher body weight), the benefits are generally modest and must be weighed against safety considerations. A review published in 2025 highlights limitations such as cross-trial comparisons and selective trial populations, emphasizing cautious interpretation of off-label regimens and the need for further data to guide practice [29].

### 4.4. Anti-IL-23

IL-23 inhibitors achieve high rates of durable clearance in psoriasis, and emerging evidence suggests they may be relatively robust across cardiometabolic subgroups, though obesity can still reduce the probability of maximal endpoints in some real-world series [21,30,31,32,33].

- Guselkumab: Real-world studies evaluating guselkumab have identified prior biologic exposure and body weight as potential determinants of treatment outcomes, with higher body weight sometimes correlating with reduced response [21,32].
- Risankizumab: Evidence on BMI impact is mixed, with some real-world series reporting reduced PASI100 rates in obese vs. non-obese patients, while others report minimal association, reflecting heterogeneity in populations, prior biologic use, and follow-up duration [21,31].
- Tildrakizumab: Multiple analyses across different body-weight strata show that higher body weight can modestly attenuate response to 100 mg dosing early in treatment, and that 200 mg may provide improved efficacy in very high body-weight groups with similar safety [30,33]. This supports individualized dose selection in patients with high body weight or high disease burden where permitted [30,33].

IL-23 inhibitors are generally strong choices in obese patients, but clinicians should anticipate that very high body weight may reduce the likelihood of PASI100 in some settings; dose optimization (where applicable) and early treat-to-target reassessment are recommended [30,31,32,33].

Data on the influence of obesity on newer oral agents, including phosphodiesterase-4 inhibitors and Janus kinase inhibitors, are emerging. While these agents may be less affected by body weight than biologics, obesity-related comorbidities may still influence safety and long-term treatment outcomes [3,23,34,35].

Table 1 summarizes obesity-specific biologics choice.

### 4.5. Small-Molecule Systemic Therapies

In contrast to monoclonal antibodies, most systemic small molecules used for psoriasis are administered as fixed oral doses and generally show less pronounced weight-dependent pharmacokinetic variability than biologics, although obesity may still influence outcomes through higher baseline inflammatory burden, comorbidities (e.g., NAFLD), adherence, and treat-to-target attainment [3,4]. Evidence on obesity as an effect modifier for small molecules is heterogeneous and molecule-specific, with several datasets suggesting minimal or no BMI effect on efficacy for apremilast, while weight-related effects have been documented for some JAK-pathway agents in clinical development programs [23,36,37].

### 4.6. Phosphodiesterase-4 Inhibition (Apremilast)

Real-world studies indicate that apremilast effectiveness in plaque psoriasis is not meaningfully influenced by BMI, with similar clinical improvements observed across BMI strata in routine practice [36,37,38]. Consistent efficacy across weight and BMI subgroups has also been reported in analyses of clinical trial and trial-like datasets, supporting the notion that apremilast response is comparatively robust to body size compared with several fixed-dose biologics [36,37].

Importantly, apremilast is frequently associated with modest weight loss, which may be clinically relevant in obese patients with psoriasis and cardiometabolic comorbidity, although weight loss is not guaranteed and is not the primary therapeutic objective [37,38,39,40].

Clinical implication: Apremilast represents a reasonable oral option in obese patients with moderate disease or contraindications to immunosuppression, particularly when an agent with BMI-stable efficacy and potential modest weight reduction is desirable [36,37,38].

### 4.7. TYK2 Inhibition (Deucravacitinib)

Phase 3 trials have established deucravacitinib efficacy versus placebo and apremilast and have characterized safety through 52 weeks and beyond [41,42,43]. While the pivotal publications were not primarily designed to test obesity–response interactions, available subgroup and real-world analyses suggest that clinically meaningful improvements are observed across BMI categories, although the evidence base remains smaller than for biologics and requires cautious interpretation [41,42,43,44].

Clinical implication: Deucravacitinib is a potent oral option for moderate-to-severe psoriasis, and current evidence supports effectiveness across patient subgroups, including those with elevated BMI; however, dedicated analyses focusing on severe obesity and long-term persistence remain an unmet need [41,42,43,44].

### 4.8. Fumarates (Dimethyl Fumarate/Fumaric Acid Esters)

Dimethyl fumarate (DMF) has demonstrated efficacy and noninferiority versus fumaric acid ester combinations in a phase 3 program, with an established titration-based dosing approach and a safety profile consistent with fumarates [45]. Real-world studies corroborate effectiveness and persistence in routine clinical settings [46,47].

However, direct evidence that obesity independently attenuates DMF clinical response is limited, and most available datasets have focused on age, tolerability, and treatment persistence rather than formal BMI-stratified efficacy endpoints [46,47,48].

Clinical implication: In obese patients, DMF remains a viable oral systemic therapy, but clinicians should consider comorbidity-driven tolerability issues (e.g., gastrointestinal adverse effects), titration adherence, and the broader cardiometabolic profile when selecting and monitoring therapy [46,48].

### 4.9. Weight Reduction as a Therapeutic Strategy

Lifestyle modification through dietary intervention and increased physical activity has been shown to improve psoriasis severity and quality of life [14,15,19,26]. Randomized controlled trials demonstrate that hypocaloric diets combined with exercise result in significant PASI reductions, particularly when used as adjuncts to systemic therapy [14,15,19,26].

Bariatric surgery provides compelling evidence for the role of weight reduction in psoriasis management [3]. Observational studies report significant improvement or remission of psoriasis following substantial weight loss, likely mediated by reduced systemic inflammation, normalization of adipokine profiles, and decreased mechanical stress [3]. Weight management should be considered an integral component of psoriasis care [14,15,19,26]. Multidisciplinary approaches involving dermatologists, primary care physicians, nutritionists, and endocrinologists are essential for addressing the complex metabolic and inflammatory needs of obese patients with psoriasis.

### 4.10. Use of GLP-1 Receptor Agonists in Obese Patients with Psoriasis

Glucagon-like peptide-1 receptor agonists (GLP-1RAs), including agents such as liraglutide, semaglutide, and the dual agonist tirzepatide, have shown promise as adjunctive therapy in psoriasis, particularly in patients with obesity or metabolic comorbidities. Recent clinical and translational studies demonstrate that GLP-1RAs can reduce psoriasis severity through both metabolic effects—such as weight loss and improved glycemic control—and direct immunomodulatory actions, including downregulation of pro-inflammatory cytokines and modulation of immune cell subsets implicated in psoriatic pathogenesis [49,50,51]. The 2025 publication by Gisondi et al. highlights tirzepatide as a potential therapeutic option for obese patients with psoriasis who are already receiving biologic therapy, suggesting a synergistic benefit in this population by targeting both metabolic and inflammatory pathways [52].

Large-scale cohort data further support the safety and efficacy of GLP-1RAs in reducing cardiovascular and psychiatric comorbidities, as well as all-cause mortality in psoriasis patients, with risk reductions more pronounced than in non-psoriatic populations [53]. However, the current evidence base is limited by small sample sizes, heterogeneity in patient populations, and confounding from concurrent therapies. While GLP-1RAs are not yet standard first-line therapy for psoriasis, they represent a promising adjunct, especially for patients with obesity or type 2 diabetes, and ongoing research is needed to clarify their optimal role and dosing in psoriasis management [49,50,51,52,53].

There are currently no large-scale randomized controlled trials or meta-analyses that directly compare glucagon-like peptide-1 receptor agonists (GLP-1RAs) to standard psoriasis therapies in patients with psoriasis. The available clinical evidence is limited to small cohort studies, retrospective analyses, and mechanistic investigations, which suggest that GLP-1RAs may reduce psoriasis severity and comorbid risks, particularly in patients with obesity or type 2 diabetes, but these studies do not provide head-to-head efficacy or safety data against established psoriasis treatments such as biologics or conventional systemic agents [49,54].

Recent reviews and cohort studies highlight the potential of GLP-1RAs as adjunctive therapy, demonstrating improvements in metabolic parameters, cardiovascular risk, and psychiatric comorbidities in psoriasis populations, with a favorable safety profile and no increase in typical adverse drug events [49]. However, these findings are not based on direct randomized comparisons with standard psoriasis therapies, and the clinical impact of GLP-1RAs as monotherapy for psoriasis remains unproven [49,54].

The medical literature emphasizes the need for well-designed, long-term randomized controlled trials to establish the comparative efficacy and safety of GLP-1RAs versus standard psoriasis treatments, optimize dosing strategies, and clarify their role in patients without metabolic comorbidities [54]. Until such data are available, GLP-1RAs should be considered primarily for psoriasis patients with coexisting obesity or diabetes, as an adjunct to conventional therapy.

## 5. Algorithm Proposal

**Figure 2:**
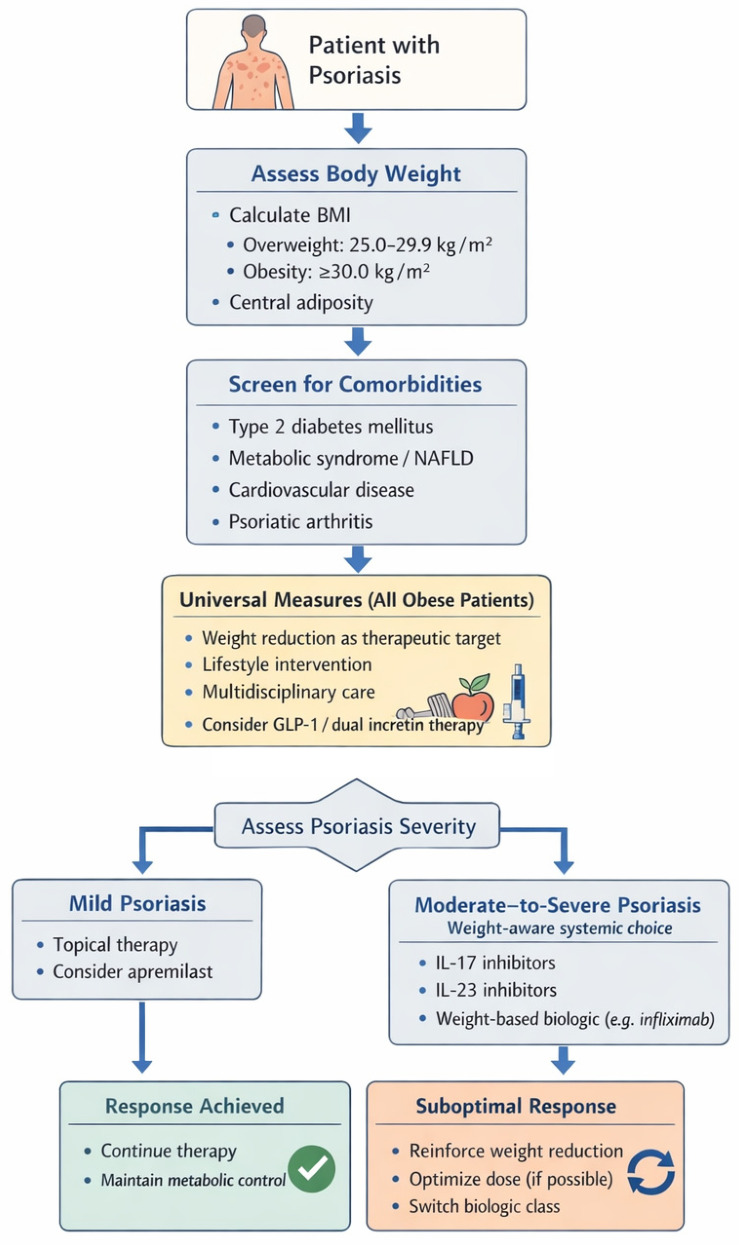
Weight-adapted therapeutic algorithm for obese patients with psoriasis.

Based on our clinical experience, and after thorough literature research, we propose a treatment algorithm for obese patients in Figure 2.

## 6. Conclusions

Future research should focus on refining risk stratification beyond BMI, incorporating measures of body composition and visceral adiposity [3]. Personalized treatment algorithms integrating metabolic status may optimize therapeutic outcomes. Further studies are also needed to clarify how emerging therapies interact with metabolic pathways and whether targeted modulation of adipose tissue inflammation can directly improve psoriasis outcomes.

Psoriasis and obesity are tightly interconnected through shared inflammatory, metabolic, and immunological pathways [2,3]. Obesity increases the risk of psoriasis, worsens disease severity, impairs therapeutic response, and amplifies systemic comorbidity burden [4,21]. Recognizing obesity as a modifiable risk factor and therapeutic target is essential for improving long-term outcomes. Integrated, multidisciplinary management strategies combining effective anti-psoriatic therapy with weight reduction and cardiometabolic risk control represent the cornerstone of modern psoriasis care.

## Figures and Tables

**Table 1:** Obesity-Specific Considerations for Biologic Selection in Psoriasis.

Biologic/Class	Obesity-Specific Consideration	Practical Implication
Anti-TNF agents	Higher BMI may reduce response, especially with fixed-dose agents.	Use when indicated, particularly in PsA, but monitor response early.
Infliximab	Weight-based dosing may partly overcome obesity-related underexposure.	Useful option in selected severely obese patients.
Ustekinumab	Has weight-adjusted dosing (90 mg if body weight > 100 kg), but high BMI may still reduce response.	Ensure correct dose; switch if targets are not met.
IL-17 inhibitors	High efficacy, although obesity may reduce PASI90/100 rates in some patients.	Good option when rapid clearance is needed.
Brodalumab/bimekizumab	Available data suggest strong efficacy across BMI groups.	Attractive options for deep clearance in obese patients.
IL-23 inhibitors	Generally durable efficacy; very high body weight may modestly reduce maximal response.	Strong long-term option; reassess early.
Tildrakizumab	Higher body weight may attenuate 100 mg response; 200 mg approved for body weight > 90 kg.	Consider dose optimization in high-weight patients.

## Data Availability

Data available upon request.

## References

[1] Boehncke W.H., Schön M.P. (2015). Psoriasis. Lancet.

[2] Davidovici B.B., Sattar N., Prinz J., Puig L., Emery P., Barker J.N., van de Kerkhof P., Ståhle M., Nestle F.O., Girolomoni G. (2010). Psoriasis and systemic inflammatory diseases: Potential mechanistic links between skin disease and co-morbid conditions. J. Investig. Dermatol..

[3] Gisondi P., Fostini A.C., Fossà I., Girolomoni G., Targher G. (2018). Psoriasis and the metabolic syndrome. Clin. Dermatol..

[4] Armstrong A.W., Harskamp C.T., Armstrong E.J. (2012). The association between psoriasis and obesity: A systematic review and meta-analysis of observational studies. Nutr. Diabetes.

[5] Setty A.R., Curhan G., Choi H.K. (2007). Obesity, waist circumference, weight change, and the risk of psoriasis in women: Nurses’ Health Study II. Arch. Intern. Med..

[6] Naldi L., Chatenoud L., Linder D., Belloni Fortina A., Peserico A., Virgili A.R., Bruni P.L., Ingordo V., Lo Scocco G., Solaroli C. (2005). Cigarette smoking, body mass index, and stressful life events as risk factors for psoriasis: Results from an Italian case-control study. J. Investig. Dermatol..

[7] Coimbra S., Oliveira H., Reis F., Belo L., Rocha S., Quintanilha A., Figueiredo A., Teixeira F., Castro E., Rocha-Pereira P. (2010). Circulating adipokine levels in Portuguese patients with psoriasis vulgaris according to body mass index, severity and therapy. J. Eur. Acad. Dermatol. Venereol..

[8] Ismail A.M.A., Saad A.E., Draz R.S. (2023). Effect of low-calorie diet on psoriasis severity index, triglycerides, liver enzymes, and quality of life in psoriatic patients with non-alcoholic fatty liver disease. Reumatologia.

[9] Morrow S., Hawkins P., Griffiths C.E.M., Tektonidis T.G., Harriss E., Scragg J., Jebb S. (2025). Impact of weight-loss interventions on psoriasis severity: A systematic review and meta-analysis. J. Eur. Acad. Dermatol. Venereol..

[10] Bellinato F., Gisondi P., Balato A., Caldarola G., Cammarata E., Campione E., Carugno A., Conti A., Corazza M., Dapavo P. (2024). Awareness of obesity among patients with psoriasis. J. Eur. Acad. Dermatol. Venereol..

[11] Karampinis E., Goudouras G., Ntavari N., Bogdanos D.P., Roussaki-Schulze A.V., Zafiriou E. (2023). Serum vitamin D levels can be predictive of psoriasis flares up after COVID-19 vaccination: A retrospective case control study. Front Med..

[12] Pavlova N.T., Kioskli K., Smith C., Picariello F., Rayner L., Moss-Morris R. (2021). Psychosocial aspects of obesity in adults with psoriasis: A systematic review. Skin. Health Dis..

[13] Nakamizo S., Honda T., Adachi A., Nagatake T., Kunisawa J., Kitoh A., Otsuka A., Dainichi T., Nomura T., Ginhoux F. (2017). High fat diet exacerbates murine psoriatic dermatitis by increasing the number of IL-17-producing γδ T cells. Sci. Rep..

[14] Ko S.H., Chi C.C., Yeh M.L., Wang S.H., Tsai Y.S., Hsu M.Y. (2019). Lifestyle changes for treating psoriasis. Cochrane Database Syst. Rev..

[15] Ford A.R., Siegel M., Bagel J., Cordoro K.M., Garg A., Gottlieb A., Green L.J., Gudjonsson J.E., Koo J., Lebwohl M. (2018). Dietary Recommendations for Adults with Psoriasis or Psoriatic Arthritis From the Medical Board of the National Psoriasis Foundation: A Systematic Review. JAMA Dermatol..

[16] Menter A., Gelfand J.M., Connor C., Armstrong A.W., Cordoro K.M., Davis D.M., Elewski B.E., Gordon K.B., Gottlieb A.B., Kaplan D.H. (2020). Joint American Academy of Dermatology–National Psoriasis Foundation guidelines of care for the management of psoriasis with systemic nonbiologic therapies. J. Am. Acad. Dermatol..

[17] Burshtein J., Armstrong A., Chow M., DeBusk L., Glick B., Gottlieb A.B., Gold L.S., Korman N.J., Lio P., Merola J. (2025). The association between obesity and efficacy of psoriasis therapies: An expert consensus panel. J. Am. Acad. Dermatol..

[18] Pongparit K., Chularojanamontri L., Limphoka P., Silpa-Archa N., Wongpraparat C. (2018). Effectiveness of and factors associated with clinical response to methotrexate under daily life conditions in Asian patients with psoriasis: A retrospective cohort study. J. Dermatol..

[19] Gisondi P., Del Giglio M., Di Francesco V., Zamboni M., Girolomoni G. (2008). Weight loss improves the response of obese patients with moderate-to-severe chronic plaque psoriasis to low-dose cyclosporine therapy: A randomized, controlled, investigator-blinded clinical trial. Am. J. Clin. Nutr..

[20] Alabas O.A., Mason K.J., Yiu Z.Z.N., Hampton P.J., Reynolds N.J., Owen C.M., Bewley A., Laws P.M., Warren R.B., Lunt M. (2023). BADBIR Study Group. Effectiveness and persistence of acitretin, ciclosporin, fumaric acid esters and methotrexate for patients with moderate-to-severe psoriasis: A cohort study from BADBIR. Br. J. Dermatol..

[21] Pirro F., Caldarola G., Chiricozzi A., Burlando M., Mariani M., Parodi A., Peris K., De Simone C. (2021). Impact of Body Mass Index on the Efficacy of Biological Therapies in Patients with Psoriasis: A Real-World Study. Clin. Drug Investig..

[22] Clark L., Lebwohl M. (2008). The effect of weight on the efficacy of biologic therapy in patients with psoriasis. J. Am. Acad. Dermatol..

[23] Hutmacher M.M., Papp K., Krishnaswami S., Ito K., Tan H., Wolk R., Valdez H., Mebus C., Rottinghaus S.T., Gupta P. (2017). Evaluating Dosage Optimality for Tofacitinib, an Oral Janus Kinase Inhibitor, in Plaque Psoriasis, and the Influence of Body Weight. CPT Pharmacomet. Syst. Pharmacol..

[24] Puig L. (2011). Obesity and psoriasis: Body weight and body mass index influence the response to biological treatment. J. Eur. Acad. Dermatol. Venereol..

[25] Yiu Z.Z.N., Mason K.J., Hampton P.J., Reynolds N.J., Smith C.H., Lunt M., Griffiths C.E.M., Warren R.B., BADBIR Study Group (2020). Drug survival of adalimumab, ustekinumab and secukinumab in patients with psoriasis: A prospective cohort study from the British Association of Dermatologists Biologics and Immunomodulators Register (BADBIR). Br. J. Dermatol..

[26] Jensen P., Zachariae C., Christensen R., Geiker N.R., Schaadt B.K., Stender S., Hansen P.R., Astrup A., Skov L. (2013). Effect of weight loss on the severity of psoriasis: A randomized clinical study. JAMA Dermatol..

[27] Hsu S., Green L.J., Lebwohl M.G., Wu J.J., Blauvelt A., Jacobson A.A. (2020). Comparable efficacy and safety of brodalumab in obese and nonobese patients with psoriasis: Analysis of two randomized controlled trials. Br. J. Dermatol..

[28] Gordon K.B., Foley P., Krueger J.G., Pinter A., Reich K., Vender R., Vanvoorden V., Madden C., White K., Cioffi C. (2021). Bimekizumab efficacy and safety in moderate to severe plaque psoriasis (BE READY): A multicentre, double-blind, placebo-controlled, randomised withdrawal phase 3 trial. Lancet.

[29] Kim D., Babaei N., Cervantes M., Gill M., Yang S., Wu J.J. (2025). Off-label biologic regimens of IL-17A inhibitors for psoriasis. J. Eur. Acad. Dermatol. Venereol..

[30] Thaci D., Piaserico S., Warren R.B., Gupta A.K., Cantrell W., Draelos Z., Foley P., Igarashi A., Langley R.G., Asahina A. (2021). Five-year efficacy and safety of tildrakizumab in patients with moderate-to-severe psoriasis who respond at week 28: Pooled analyses of two randomized phase III clinical trials (reSURFACE 1 and reSURFACE 2). Br. J. Dermatol..

[31] Blauvelt A., Leonardi C.L., Gooderham M., Papp K.A., Philipp S., Wu J.J., Igarashi A., Flack M., Geng Z., Wu T. (2020). Efficacy and Safety of Continuous Risankizumab Therapy vs Treatment Withdrawal in Patients With Moderate to Severe Plaque Psoriasis: A Phase 3 Randomized Clinical Trial. JAMA Dermatol..

[32] Reich K., Papp K.A., Armstrong A.W., Wasfi Y., Li S., Shen Y.K., Randazzo B., Song M., Kimball A.B. (2019). Safety of guselkumab in patients with moderate-to-severe psoriasis treated through 100 weeks: A pooled analysis from the randomized VOYAGE 1 and VOYAGE 2 studies. Br. J. Dermatol..

[33] Dapavo P., Burlando M., Guarneri C., Megna M., Narcisi A., Talamonti M., Gisondi P. (2024). Tildrakizumab: The value of a personalized and flexible approach for treating moderate-to-severe plaque psoriasis in patients with high body weight or high disease burden. Expert Opin. Biol. Ther..

[34] Ighani A., Georgakopoulos J.R., Yeung J. (2020). Tofacitinib for the treatment of psoriasis and psoriatic arthritis. G. Ital. Dermatol. Venereol..

[35] Valenzuela F., Paul C., Mallbris L., Tan H., Papacharalambous J., Valdez H., Mamolo C. (2016). Tofacitinib versus etanercept or placebo in patients with moderate to severe chronic plaque psoriasis: Patient-reported outcomes from a Phase 3 study. J. Eur. Acad. Dermatol. Venereol..

[36] Malara G., Politi C., Trifirò C., Verduci C., D’Arrigo G., Testa A., Tripepi G. (2021). Effectiveness of Apremilast in Real Life in Patients with Psoriasis: A Longitudinal Study. Acta Derm. Venereol..

[37] Cavanaugh C., Orroth K., Qian X., Kumparatana P., Klyachkin Y., Colgan S., Cordey M. (2024). Diabetes and obesity burden and improvements in cardiometabolic parameters in patients with psoriasis or psoriatic arthritis receiving apremilast in a real-world setting. JAAD Int..

[38] Ferguson L.D., Cathcart S., Rimmer D., Semple G., Brooksbank K., Paterson C., Brown R., Harvie J., Gao X., Radjenovic A. (2022). Effect of the phosphodiesterase 4 inhibitor apremilast on cardiometabolic outcomes in psoriatic disease-results of the Immune Metabolic Associations in Psoriatic Arthritis study. Rheumatology.

[39] Hansildaar R., van Geel E.H., Çoban F., Dijkshoorn B., Heslinga M., Bos R., Korteweg M.A., van Kuijk A.W.R., Nurmohamed M.T. (2025). Cardiometabolic Effects of Apremilast in Patients With Psoriatic Arthritis: A Prospective Cohort Study. J. Rheumatol..

[40] Mehta P., Kharouf F., Abarza V.C., Gao S., Cook R.J., Poddubnyy D., Gladman D.D., Chandran V. (2025). Exploring the impact of conventional and targeted DMARDs on body weight in patients with PsA. Rheumatology.

[41] Armstrong A.W., Gooderham M., Warren R.B., Papp K.A., Strober B., Thaçi D., Morita A., Szepietowski J.C., Imafuku S., Colston E. (2023). Deucravacitinib versus placebo and apremilast in moderate to severe plaque psoriasis: Efficacy and safety results from the 52-week, randomized, double-blinded, placebo-controlled phase 3 POETYK PSO-1 trial. J. Am. Acad. Dermatol..

[42] Armstrong A.W., Lebwohl M., Warren R.B., Sofen H., Imafuku S., Ohtsuki M., Spelman L., Passeron T., Papp K.A., Kisa R.M. (2025). Safety and Efficacy of Deucravacitinib in Moderate to Severe Plaque Psoriasis for Up to 3 Years: An Open-Label Extension of Randomized Clinical Trials. JAMA Dermatol..

[43] Strober B., Blauvelt A., Warren R.B., Papp K.A., Armstrong A.W., Gordon K.B., Morita A., Alexis A.F., Lebwohl M., Foley P. (2024). Deucravacitinib in moderate-to-severe plaque psoriasis: Pooled safety and tolerability over 52 weeks from two phase 3 trials (POETYK PSO-1 and PSO-2). J. Eur. Acad. Dermatol. Venereol..

[44] Okabe Y., Hagino T., Takahashi Y., Saeki H., Fujimoto E., Kanda N. (2026). Two-Year Real-World Effectiveness of Deucravacitinib 6 mg in Psoriasis: A Single-Center Analysis Stratified by Body Mass Index or Age in a Japanese Cohort. J. Dermatol..

[45] Mrowietz U., Szepietowski J.C., Loewe R., van de Kerkhof P., Lamarca R., Ocker W.G., Tebbs V.M., Pau-Charles I. (2017). Efficacy and safety of LAS41008 (dimethyl fumarate) in adults with moderate-to-severe chronic plaque psoriasis: A randomized, double-blind, Fumaderm^®^—And placebo-controlled trial (BRIDGE). Br. J. Dermatol..

[46] Gnesotto L., Mioso G., Bardazzi F., Filippi F., Di Lernia V., Motolese A., Di Nuzzo S., Conti A., Arginelli F., Corazza M. (2023). Dimethyl Fumarate Treatment in Patients with Moderate-to-Severe Psoriasis: A 52-week Real-life Study. Acta Derm. Venereol..

[47] Maul L.V., Ak M., Cerminara S.E., Simic D., Gössinger E.V., Roider E., Darzina A., Zink A., Oyanguren Monferrer I., Oestereich F. (2025). Dimethyl Fumarate Treatment for Psoriasis in Real-World Clinical Practice: An Analysis from the Swiss Registry. Dermatology.

[48] van de Kerkhof P.C.M., Loewe R., Mrowietz U., Falques M., Pau-Charles I., Szepietowski J.C. (2020). Quality of life outcomes in adults with moderate-to-severe plaque psoriasis treated with dimethylfumarate (DMF): A post hoc analysis of the BRIDGE study. J. Eur. Acad. Dermatol. Venereol..

[49] Atiquzzaman N., Razdolsky N., Parmar M.S. (2025). GLP-1 receptor agonists: Emerging therapeutic potential in psoriasis management—Current evidence and future outlook. Eur. J. Clin. Pharmacol..

[50] Paschou I.A., Sali E., Paschou S.A., Psaltopoulou T., Nicolaidou E., Stratigos A.J. (2025). The effects of GLP-1RA on inflammatory skin diseases: A comprehensive review. J. Eur. Acad. Dermatol. Venereol..

[51] Buysschaert M., Baeck M., Preumont V., Marot L., Hendrickx E., Van Belle A., Dumoutier L. (2014). Improvement of psoriasis during glucagon-like peptide-1 analogue therapy in type 2 diabetes is associated with decreasing dermal γδ T-cell number: A prospective case-series study. Br. J. Dermatol..

[52] Gisondi P., Brigenti N., Bellinato F., Girolomoni G. (2025). Tirzepatide in obese patients with psoriasis on biological therapy: Is this a window of opportunity?. J. Eur. Acad. Dermatol. Venereol..

[53] Olbrich H., Kridin K., Zirpel H., Hernandez G., Sadik C.D., Gaffal E., Thaçi D., Ludwig R.J. (2026). Glucagon-like peptide-1 receptor agonists and reduced mortality, cardiovascular and psychiatric risks in patients with psoriasis: A large-scale cohort study. Br. J. Dermatol..

[54] Bilgin E., Venerito V., Bogdanos D.P. (2025). Glucagon-Like Peptide-1 (GLP-1) receptor agonists in rheumatology: A review of current evidence and future directions. Autoimmun. Rev..

